# 

**Published:** 1855-06

**Authors:** 


### A DELUGE OF BOOKS.

After reading the subjoined list of new books that are just ready to issue from the press, or are in process of preparation, no one can doubt that the profession is being thoroughly inoculated with the *cacœthes scribendi.* If many of the new works are of the size of some of very recent issue that are now lying on our table we cannot, for our part, see how a physician who has any number of daily visits to pay will ever have time to read more than their table of contents. The following are announced:—

By Lindsay & Blakiston:—Canton's *Pathological and Surgical Observations;* Dixon's *Guide to the Practical Study of Diseases of the Eye;* Jones' *Pathological and Clinical Observations on the Morbid Conditions of the Stomach;* Lee's *Pathological and Surgical Observations;* Garrod on the *Essentials of Materia Medica;* Johnson on *Epidemic Cholera and Diarrhea.*

By Blanchard & Lea:—La Roche on *Yellow Fever;* Prof. Dickson, of Charleston, on *the Elements of Medicine;* Browne on *Surgical Diseases of Females;* Graham's *Elements of Chemistry*; Ludlow's *Manual of Examinations*, a new edition; Lehman's *Physiological Chemistry;* Tyler Smith on *Leucorrhœa;* Rokitansky's *Pathological Anatomy;* Gross's *System of Surgery;* Carpenter on the *Microscope and its Revelations;* Todd and Bowman's *Physiological Anatomy and Physiology of Man;* Hoblyn's *Dictionary of Medical Terms;* Sibson's *Medical Anatomy; Manual of the Practice of Medicine*, by Barlow; *General Physiology*, by Carpenter; Toynbee's *Aural Surgery;* Curling on *Diseases of the Testis*, new edition; Peaselee's *Human Histology;* Longet's *Treatise on Physiology;* Stillé's *Principles of Therapeutics.*

By S. S. & W. Wood:—*Diseases of the Rectum*, by Richard Quain; *A Series of Anatomical Plates*, by Jones Quain; *The Anatomical Remembrancer.*

Dr. Stephen Smith, of New York, is engaged upon a work entitled *Medical Jurisprudence in its Application to the Practice of Medicine, Surgery, and Midwifery in the United States.* Prof. Draper, of New York, is preparing a *Treatise on Physiology*, which will be issued in two or three months; Prof. Mott's *Tour in the East*, will soon appear in a second edition.

D. W. Y.

SERIES FOR 1855.

### The Western Journal

OF

### MEDICINE AND SURGERY.

L. P. YANDELL, M. D., EDITOR.

The first Number of the Thirty-first Volume is now before the public, dated January, 1855. New subscribers can be supplied from this date. No Physician's library is completed, we may venture to say, without the

**WESTERN JOURNAL,**

Which besides being a chronicle of Western Medical Information, abounds in matter calculated to interest every Physician.

The Editor has filled the position he occupies in relation to it, for ten years. It was the first Medical Journal in the West for years, and now ranks among the first in the Union.

**A SERIES OF LECTURES**

*On Inflammation and Ulceration of the Cervix Uteri*, by HENRY MILLER, Professor of Obstetrics in the Unveirsity of Louisville, opens the January Number for 1855, and the same will be continued in the two succeeding Numbers.

☞ All communications, containing subscription or any other business matter connected with the publishing department, should be addressed to

J. F. BRENNAN, Louisville, Ky.

And all pertaining to the editorial department, to the Editor, L. P. YANDELL, M. D., Louisville, Ky.

☞ Subscription, *Three Dollars* per annum. No name entered until the money is received.

SERIES FOR 1855.

### The Western Journal

OF

### MEDICINE AND SURGERY.

L. P. YANDELL, M. D., EDITOR.

The second Number of the Thirty-first Volume is now before the public, dated January, 1855. New subscribers can be supplied from this date. No Physician's library is completed, we may venture to say, without the

**WESTERN JOURNAL,**

Which besides being a chronicle of Western Medical Information, abounds in matter calculated to interest every Physician.

The Editor has filled the position he occupies in relation to it, for ten years. It was the first Medical Journal in the West for years, and now ranks among the first in the Union.

**A SERIES OF LECTURES**

*On Inflammation and Ulceration of the Cervix Uteri*, by HENRY MILLER, Professor of Obstetrics in the Unveirsity of Louisville, opens the January Number for 1855, and the same will be continued in the two succeeding Numbers.

☞ All communications, containing subscription or any other business matter connected with the publishing department, should be addressed to

J. F. BRENNAN, Louisville, Ky.

And all pertaining to the editorial department, to the Editor, L. P. YANDELL, M. D., Louisville, Ky.

☞ Subscription, *Three Dollars* per annum. No name entered until the money is received.

### SERIES FOR 1855.

### The Western Journal

OF

### MEDICINE AND SURGERY.

L. P. YANDELL, M. D., EDITOR.

The second Number of the Thirty-first Volume is now before [illegible] dated January, 1855. New subscribers can be supplied [illegible]. No Physician's library is completed, we may venture to [illegible]

**WESTERN JOURNAL,**

Which besides being a chronicle of Western Medical information, abounds in matter calculated to interest every Physician.

The Editor has filled the position he occupies in relation to it, for ten years. It was the first Medical Journal in the West [illegible] years, and now ranks among the first in the Union.

**A SERIES OF LECTURES**

*On Inflammation and Ulceration of the Cervix Uteri*, by HENRY MILLER, Professor of Obstetrics in the Unveirsity of Louisville, opens the January Number for 1855, and the same will be continued in the two succeeding Numbers.

☞ All communications, containing subscription or any other business matter connected with the publishing department, should be addressed to

J. F. BRENNAN, Louisville, Ky.

And all pertaining to the editorial department, to the Editor, L. P. YANDELL, M. D., Louisville, Ky.

☞ Subscription, *Three Dollars* per annum. No name entered unti the money is received.

